# Brentuximab vedotin combined with cisplatin, cytarabine, and dexamethasone treatment in transplant-eligible Korean patients with relapsed or refractory Hodgkin’s lymphoma

**DOI:** 10.1007/s44313-025-00097-z

**Published:** 2025-11-21

**Authors:** Jun Ho Yi, Young Hoon Park, Myung-won Lee, Kwai Han Yoo, Junshik Hong, Hyeon-Seok Eom

**Affiliations:** 1 Division of Hematology-Oncology, Department of Medicine, Samsung Medical Center, Sungkyunkwan University School of Medicine, Seoul, Korea; 2 Division of Hematology-Oncology, Department of Internal Medicine, Ewha Womans University College of Medicine, Seoul, Korea; 3 Division of Hematology and Oncology, Department of Internal Medicine, Chungnam National University Hospital, Daejeon, Republic of Korea; 4 Division of Hematology-Oncology, Gachon University College of Medicine, Incheon, Korea; 5 Division of Hematology-Oncology, Department of Internal Medicine, Seoul National University Hospital, Seoul, Korea; 6 Center for Hematologic Malignancies, National Cancer Center, 323 Ilsan-Ro, Ilsandong-Gu, Goyang, Gyeonggi-Do 10408 Korea

**Keywords:** Hodgkin's lymphoma, Autologous stem cell transplantation, Brentuximab vedotin

## Abstract

**Purpose:**

Brentuximab vedotin (BV)-based combinations have shown promising results in patients with relapsed or refractory Hodgkin’s lymphoma (RRHL) in Western countries. We conducted a phase II study to further define the role of BV-based salvage therapy in transplant-eligible patients with RRHL in Korea.

**Methods:**

Transplant-eligible patients with RRHL were recruited to receive two cycles of BV (1.8 mg/kg) plus DHAP (cisplatin 100 mg/m^2^, cytarabine 2 g/m^2^, and dexamethasone 40 mg). Autologous stem cells were collected from non-progressive patients who received one more cycle of BV-DHAP. After three cycles, the responding patients underwent autologous stem cell transplantation (ASCT). The primary endpoint was a complete metabolic response (CMR) rate after two cycles of BV-DHAP.

**Results:**

Between April 2022 and August 2024, seven lymphoma cases were recruited. Owing to slow accrual, the study was terminated early. Their median age was 30 years (range, 24–62 years). All patients received at least two cycles of BV-DHAP; the overall response rate (ORR) was 100%, and four patients showed CMR (57%). One patient withdrew consent after the second cycle, and the other six patients received one more cycle of BV-DHAP, resulting in five patients achieving CMR (71%). One patient failed to receive ASCT, and all five patients who received ASCT achieved post-transplant CMR with a 80% complete response (CR) over a 12-month duration. Thrombocytopenia was the most common grade 3 or higher grade hematologic adverse events (*n* = 5). Grade 2 nausea occurred in three patients. Three patients experienced dose reduction, and four patients experienced treatment delays.

**Conclusions:**

Despite its encouraging early efficacy, the administration of BV-DHAP in Korean patients with RRHL requires careful monitoring due to toxicity concerns.

**Supplementary Information:**

The online version contains supplementary material available at 10.1007/s44313-025-00097-z.

## Introduction

Patients with classical Hodgkin’s lymphoma (HL) are usually treated with front-line combination chemotherapy with doxorubicin, bleomycin, vinblastine, and dacarbazine (ABVD). However, approximately 10–30% of patients suffer from relapsed or refractory disease [1], and the current standard of care is intensified salvage chemotherapy followed by autologous stem cell transplantation (ASCT) [2]. However, conventional salvage regimens fail to achieve long-term remission in approximately 50% of the patients [3], which leaves a significant unmet need for these patients. Multiple studies have demonstrated that achieving a positron emission tomography-computed tomography (PET-CT)-negative complete metabolic response (CMR) before ASCT is the strongest predictor of post-transplant long-term outcomes [4, 5].

Brentuximab vedotin (BV) is an antibody–drug conjugate in which monomethyl auristatin E covalently binds to monoclonal chimeric antibodies against CD30 expressed on the surface of malignant Reed-Sternberg cells. As MMAE is freed from intracellular lysosomes, it acts as an anti-microtubule agent and induces apoptosis in Reed-Sternberg cells. BV monotherapy has demonstrated valuable clinical outcomes and manageable toxicities in pivotal studies [6, 7] and real-world reports [8] on relapsed or refractory HL (RRHL). Subsequently, several BV-based salvage combinations in HL evaluated in phase 2 trials have shown a CMR rate of over 70%, which is considerably higher than that of chemotherapy alone [9]. Thus, BV-based salvage regimens are considered the standard of care for patients with RRHL who are eligible for ASCT [10]. However, the efficacy and safety of BV-based salvage regimens have not been adequately assessed in Asia owing to the rarity of the disease [11] and limited accessibility of the drug.

To further define the role of BV-based salvage therapy in transplant-eligible patients with RRHL in Asia, we conducted a phase 2 study to evaluate the efficacy and safety of BV combined with cisplatin, cytarabine, and dexamethasone (BV-DHAP) as an induction treatment before ASCT.

## Patients and methods

### Patients

This multicenter phase 2 study recruited patients aged 19–70 years with a documented diagnosis of RRHL. RRHL was defined as follows: 1) Deauville score (DS) of 5 on PET-CT after two or three cycles of ABVD treatment; 2) DS 4 to 5 after the completion of ABVD treatment or radiotherapy and not candidates for involved site radiation therapy; 3) Radiologically confirmed relapse after achieving CMR. If BV was used as the first-line treatment, the subject could be registered only if recurrence was confirmed after six months. At least one measurable lesion and appropriate organ function are required to tolerate treatment and ASCT. Patients with non-Hodgkin’s lymphoma or nodular lymphocyte-predominant Hodgkin’s lymphoma were excluded. Patients with confirmed CNS involvement who had already received two or more prior lines of therapy were also excluded. Other inclusion and exclusion criteria are detailed in the Supplementary Materials.

### Procedures

**Fig. 1:**
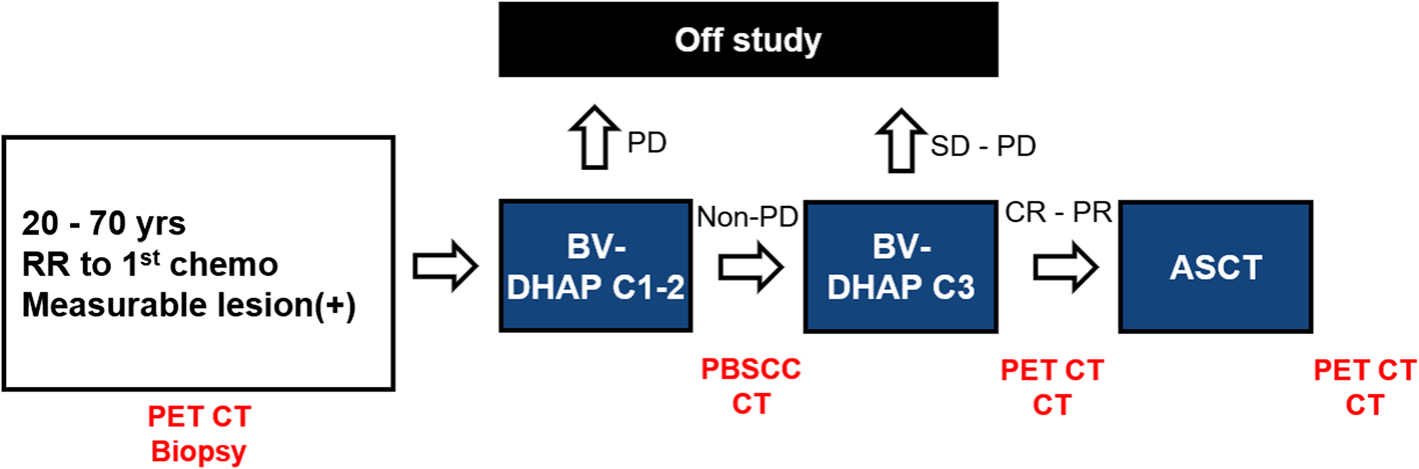
Process of the study (RR, relapse or refractory; BV, brentuximab vedotin; DHAP, cisplatin, cytarabine, and dexamethasone; PET-CT, positron emission tomography–computed tomography; PBSCC, peripheral blood stem cell collection; CR, complete response; PR, partial response; SD, stable disease; PD, progressive disease; ASCT, autologous stem cell transplantation)

The induction regimen comprised BV (1.8 mg/kg, day 1) plus cisplatin (75 mg/m^2^, continuous infusion over 24 h, day 1), cytarabine (1,500 mg/m^2^, twice a day, day 2), and dexamethasone (40 mg, orally, days 1–4) administered every three weeks. Patients were evaluated after two cycles of induction treatment, and those without a progressive disease (PD) received one additional cycle of treatment. After three treatments, patients who achieved a complete response (CR) or partial response (PR) proceeded to receive ASCT, which was performed in accordance with the protocol as per the site’s policy. If PD was observed during any response evaluation or a response was not achieved after three cycles of treatment, the patient was withdrawn from the study. Pegylated granulocyte-colony stimulating factor (G-CSF) was routinely administered after the first and third cycles. Peripheral blood stem cells were collected after two cycles. These procedures are described in Fig. 1, and the adjustment of the dosage and dosing schedule is provided in the Supplementary Material. The study was registered at ClinicalTrials.gov (NCT05243693).

### Statistical considerations

The primary endpoint of the current study was CMR after two cycles of BV-DHAP. The secondary endpoints were the overall response rate (ORR), progression-free survival (PFS), overall survival (OS), and safety profile. Response was evaluated according to the Lugano criteria based on CT and PET-CT [12]. PFS was calculated from the date of the first administration of the study drug to the date of disease progression or death from any cause. OS was calculated from the date of the first administration of the study drug to the date of death from any cause, and was censored at the date of the last visit for patients whose deaths could not be confirmed. PFS and OS were estimated using the Kaplan–Meier method. The safety profile was assessed using the National Cancer Institute Common Terminology Criteria for Adverse Events (version 5.0).

For sample size calculation, the null hypothesis was that the CMR of DHAP would be 30%, and the alternative hypothesis was that the CMR would be 60% with the addition of BV [13]. Based on a type 1 error rate of 0.05 and power of 90%, 25 eligible cases were evaluated using a single-stage phase 2 study design [14]. Assuming a 10% loss rate, 30 patients were enrolled in this study. When CMR was achieved in at least 12 patients, the null hypothesis was rejected. All statistical analyses were performed using the software IBM SPSS Statistics for Windows (version 21.0; IBM Corp., Armonk, NY, USA).

### Compliance with ethical standards

The authors declare no conflicts of interest. All procedures involving human participants were performed in accordance with ethical standards of the institutional and/or national research committee and the Declaration of Helsinki or comparable ethical standards. This study was approved by the institutional review boards of the respective institutions (approval numbers; NCC 2020–0293; CNUH 2021–12-070; EUMC 2021–12-044; GCIRB 2022–089; J-2201–055-1290).

## Results

### Patients’ characteristics

Between April 2022 and August 2024, seven consecutive patients were recruited. Owing to slow accrual, the study was terminated early. Their median age was 30 years (range, 24–62 years), and five patients (71%) were male. At the time of initial diagnosis, five patients had stage 2 disease and two patients had stage 4 disease. Three patients had B symptoms, and one patient had both bulky disease and extranodal involvement. All patients received ABVD as frontline treatment, with a median cycle of 4 (range, 4–6 cycles), and no patient received radiation therapy.

Patients participated in the study after a median duration of 12.8 months (95% confidence interval (CI), 1.0–24.6) after completion of ABVD treatment. Four patients had relapsed disease after achieving CMR, while the other three patients had DS 4–5 after the completion of ABVD treatment. At the time of relapse, bulky disease, extranodal involvement, and bone marrow involvement were present in one patient, whereas serum lactate dehydrogenase levels were elevated in three patients; the details are presented in Table 1.

**Table 1:** Characteristics at the baseline and time of relapse (*N* = 7)

	***N* (%)**
**Baseline & 1 L treatment**	
Age (median, range)	30 (24–62)
Male	5 (71)
Ann Arbor stage	
II IV	5 2
Presence of	
Bulky disease B Symptoms Extranodal involvement	1 (14) 3 (42) 1 (14)
1 L regimen	
ABVD Radiation treatment	7 (100) 0 (0)
Cycles of the 1 L treatment (median, range)	4 (4–6)
Best response to the 1 L treatment	
CR PR PD	5 (71) 1 (14) 1 (14)
**At the time of relapse**	
Relapse features	
Relapses after achieving CMR DS 4–5 after completion of the 1 L treatment	4 (57) 3 (42)
Median duration of response from the 1 L treatment	12.8 months (95% CI, 1.0–24.6)
ECOG PS 0/I/II	2/4/1
Presence of	
Bulky disease B Symptoms Extranodal involvement Bone marrow involvement Elevated serum LDH	1 (14) 0 (0) 1 (14) 1 (14) 3 (42)
FDG uptake according to the DS	
4 5	1 (14) 6 (86)

### Treatment course and outcomes of BV-DHAP

**Fig. 2:**
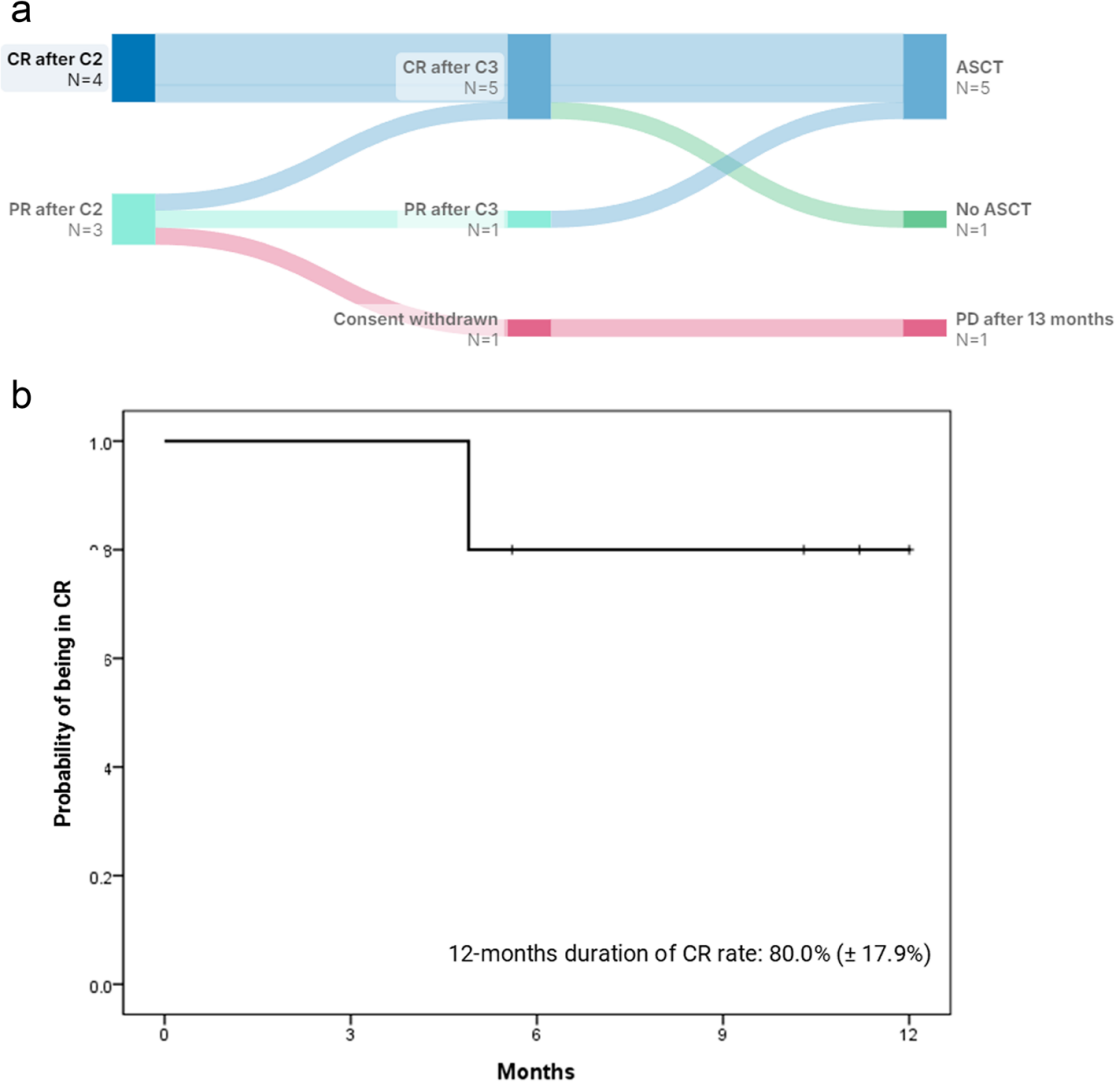
Courses of patients (**A**) and the duration of complete response (**B**)

All patients received at least two cycles of BV-DHAP, resulting in an ORR of 100% and a CR rate of 57% (4/7). One patient who achieved a PR withdrew consent after the second cycle and refused further treatment. The patient eventually developed PD after three months and died of the disease 13 months later. The other six patients completed three cycles of BV-DHAP. Among them, one patient could not undergo ASCT because of prolonged grade 4 thrombocytopenia. Stem cell collection was successful in the remaining five patients who did not receive plerixafor. They received ASCT; two patients received busulfan/cyclophosphamide/etoposide, two received busulfan/etoposide/melphalan, and one received bendamustine/etoposide/cytarabine/melphalan. After ASCT, all patients achieved CR, but one patient who had an elevated baseline LDH level quickly progressed after 4.9 months. The patient’s clinical course is shown in Fig. 2A. The median duration of CR was not reached, and the 12-month sustained CR rate was 80.0% (± 17.9%) (Fig. 2B).

The most common grade 3 or higher grade hematologic adverse events (AEs) were thrombocytopenia (*n* = 5), which led to a dose reduction of cisplatin. None of the patients had febrile neutropenia. In terms of grade 3 or higher grade non-hematologic AEs, one patient experienced a grade 4 anaphylactic event during BV infusion during the second cycle. Subsequently, the patient received DHAP alone during the third cycle. No cases of pulmonary dysfunction occurred during the study period. Grade 2 nausea occurred in three patients. Two patients had reduced cisplatin doses owing to persistent nausea, diarrhea, and creatinine elevation. Four patients experienced treatment delays owing to nausea (*n* = 2), thrombocytopenia (*n* = 1), or creatinine elevation (*n* = 1).

## Discussion

The introduction of novel anti-lymphoma agents, including BV and immune checkpoint inhibitors (ICIs), has changed the treatment landscape for RRHL and substantially improved the percentage of patients achieving quality remission. However, consolidative ASCT remains the standard of care because of its proven long-term efficacy [15]. Because a deep lymphoma response reflected by PET-CT-negative CMR is a well-known surrogate marker for long-term remission before ASCT, we determined CMR as the endpoint of the current study.

Although BV monotherapy in RRHL results in CR rates of approximately 30% [16], several BV-based combinations have been investigated as salvage regimens before ASCT. In the phase 2 BRESELIBET trial, the GELTAMO group evaluated the efficacy of BV plus ESHAP (etoposide, methylprednisolone, cytarabine, and cisplatin) versus ESHAP alone [17]. BV-ESHAP was associated with a significantly higher CMR (69.7% vs. 48.0%, *P* = 0.007) without additional toxicity profiles. In a US single-arm study, BV combined with ICE (ifosfamide, carboplatin, and etoposide) demonstrated a CR rate of 74% and a 5-year PFS rate of 77% [18]. LaCasce et al. conducted a single-arm study using BV plus bendamustine, and the ORR and CR rates were 92.5% and 73.6%, respectively [19]. In a study conducted by the HOVON group, the CMR rate was 81% with BV-DHAP [20]. Collectively, BV-based combinations generate CR rates of approximately 70%, which is higher than those of monotherapy. In our study, five out of seven patients (71%) achieved CR after three cycles of salvage therapy.

Despite the improved outcomes of BV-based combinations, there have been no such studies in Asia, probably due to the lower incidence of HL in this region [21]. Likewise, most Asian prospective BV studies recruited a smaller number of patients compared with Western studies [22]. In addition to the lower incidence of HL, patient enrollment was lower than expected because the current study required eligibility for ASCT. Because HL occurs more frequently in young patients, we observed an increased number of patients who chose prolonged therapy with ICIs over ASCT, which is recognized to cause significant infertility.

The numerical data obtained in this study are satisfactory. The CMR after cycle 2 was 51%, and five of the seven patients achieved CMR after cycle 3. Five of the seven patients could proceed with ASCT, with a CR rate of 80% over a 12-month duration. However, owing to the limited number of patients, it is difficult to conclude whether BV-DHAP is a superior regimen. In addition, DHAP is not generally tolerated, as patients require relatively frequent dose reductions and treatment delays during only three cycles of treatment.

In conclusion, our findings suggest that the BV-based combination of BV-DHAP induces a high PET-negative CMR without interfering with stem cell collection, enabling successful ASCT. Therefore, large-scale studies combining more tolerable regimens are required.

## Supplementary Information

Supplementary Material 1.

## Data Availability

No datasets were generated or analysed during the current study.
