# Supplementary material for: Brentuximab vedotin combined with cisplatin, cytarabine, and dexamethasone treatment in transplant-eligible Korean patients with relapsed or refractory Hodgkin’s lymphoma
Source: Blood Res. 2025 Nov 21;60(1):62. doi: 10.1007/s44313-025-00097-z (PMC12638574; doi:10.1007/s44313-025-00097-z)
Supplement: Supplementary file 1 — Supplementary Material 1. [file 44313_2025_97_MOESM1_ESM.docx]

**1. Dose and schedules**

After screening process, patients who are qualified for entry into this study will initiate treatment. Treatment should be initiated within 21 days after ICF acquisition. If weight change between cycles is ≥ ± 10% compared to baseline body weight measured at the first dosing visit of induction therapy, the dosage of the study drugs can be appropriately adjusted.

| Study Drug | Dosage | Dosing Date |
| --- | --- | --- |
| **Brentuximab vedotin** | 1.8 mg/kg IV over 30 minutes | D1 |
| **Cisplatin** | 100 mg/m2 + NS 1000 mL CIV over 24 hours | D1 |
| **Cytarabine** | 2.0 g/m2 + 5% DW 250 mL IV over 3 hours twice a day | D2 |
| **Dexamethasone** | 40 mg IV or PO | D1-4 |

- If there is no contraindication, pegylated G-CSF will be administered between D3 and 5 (cycle 1&3)

- PBSCC will be performed in subjects with no progressive disease (PD) after the completion of 2 cycles. Mobilization will be carried according to the participating site's protocol.

- Administration of antiemetics, pneumocystis carinii pneumonia (PCP) prophylaxis, and other conservative management will be carried out according to the policy of participating site.

## 2. Adjustment of Dosage and Dosing Schedule

- ANC < 1.5 x 10^9^/L or platelet < 100 x 10^9^/L: Induction therapy will be delayed for 1 week.

- If the level resolves to ANC ≥ 1.5 x 10^9^/L and platelet ≥ 100 x 10^9^/L after 1 week, induction therapy is implemented without dose reduction.

- If the level does not resolve to ANC ≥ 1.5 x 10^9^/L and platelet ≥ 100 x 10^9^/L after 1 week, induction therapy is additionally postponed for 1 week. In this case, conservative treatment such as blood transfusion for recovery promotion may be performed at the discretion of the investigator. Such treatment can be executed up to a total of 2 times.

- If treatment delay for 3 weeks or more is necessary, withdrawal of the subject from this study is considered.

- If treatment delay occurs for 2 weeks or more due to a haematological toxicity from the previous cycle, dose of cisplatin is reduced by 25%. If treatment delay occurs due to a haematological toxicity even after the dose reduction, dose of cytarabine is reduced by 25%.

- If Grade 3 or higher sensory/motor neuropathy occurs, dose reduction of cisplatin is considered.

- In the case of other haematological or non-haematological toxicities, dose reduction or treatment delay is implemented at the discretion of the investigator.

**3. Study eligibility**

## 3-1. Inclusion Criteria

A subject should meet all of the following criteria prior to entry into the study:

1. Histologically confirmed diagnosis of classical Hodgkin’s lymphoma. CD30 has to be positive

2. Refractory to the first-line treatment or relapse after the first-line treatment (radiologically confirmed)

- Deauville score 5 as a result of the restaging PET-CT after 2 to 3 cycles of ABVD treatment

- Deauville score 4 to 5 even after the completion of ABVD treatment or radiotherapy and are not candidates for ISRT (involved site radiation therapy)

- Radiologically confirmed relapsed after achieving CR

- If Brentuximab Vedotin (BV) is used as the first-line treatment, the subject could be registered only if recurrence is confirmed after 6 months.

3. At least one measurable lesion(s)

- nodal lesion longest transverse diameter (LDi) ≥ 1.5 cm;

- extranodal lesion LDi ≥ 1.0 cm

4. Age between 19 and 70

5. ECOG PS 0 ~ 2

6. Appropriate organ functions to tolerate the protocol treatment and ASCT

Absolute Neutrophil Count (ANC) ≥ 1.0 x 10^9/L

Platelets ≥ 75 x 10^9/L

Hemoglobin ≥ 8.0 g/dL

Serum Creatinine ≤ 1.5 x upper limit normal (ULN)

Serum Bilirubin ≤ 1.5 x ULN

AST and ALT ≤ 3 x ULN

Corrected diffusing capacity for carbon monoxide (DLCO) ≥50 percent

7. Female patient is either post-menopausal for at least 1 year before the screening visit or surgically sterile or if of childbearing potential, agree to practice 2 effective methods of contraception, at the same time, from the time of signing the informed consent through 6 months after the last dose of study drug, or agrees to completely abstain from heterosexual intercourse

8. Male patients, even if surgically sterilized, (i.e., status post vasectomy) agree to practice effective barrier contraception during the entire study period and through 6 months after the last dose of study drug, or agrees to completely abstain from heterosexual intercourse

9. Written informed consent

## Exclusion Criteria

Any subject who meets any of the following criteria will not qualify for entry into the study:

1. Non-Hodgkin’s lymphoma or nodular lymphocyte predominant Hodgkin’s lymphoma

2. 2 or more prior lines of treatment (Palliative radiotherapy or high-dose steroid therapy for symptom control are allowed)

3. Known cerebral or meningeal disease (HL or any other etiology), including signs or symptoms of progressive multifocal leukoencephalopathy

4. Confirmed CNS involvement and/or symptomatic neurologic disease compromising normal activities of daily living or requiring medications

5. Patients who cannot tolerate high-dose therapy followed by ASCT described in the inclusion criteria 6

6. Patients with severe or uncontrolled medical conditions, abnormal laboratory findings, or psychiatric disorders. For example,

i. severely impaired pulmonary function as defined as spirometry and DLCO (diffusing capacity of the lung for carbon monoxide) that is 50% or less of the normal predicted value and/or O2 saturation that is 90% or less at rest on room air

ii. any active (acute or chronic) or uncontrolled infection/disorders that impair the ability to evaluate the patient or for the patient to complete the study

iii. nonmalignant medical illnesses that are uncontrolled or whose control may be jeopardized by this study drug, such as severe hypertension that is not controlled with medical management and thyroid abnormalities when thyroid function cannot be maintained in the normal range by medication

iv. creatinine clearance < 30 mL/min

7. Known history of any of the following cardiovascular conditions

i. Myocardial infarction within 2 years of enrollment

ii. New York Heart Association (NYHA) Class III or IV heart failure

iii. Evidence of current uncontrolled cardiovascular conditions, including cardiac arrhythmias, congestive heart failure (CHF), angina, or electrocardiographic evidence of acute ischemia or active conduction system abnormalities

iv. Recent evidence (within 6 months before first dose of study drug) of a left-ventricular ejection fraction <50%

8. Synchronous or metachronous malignant tumor other than HL within 5 years (except for adequately treated basal cell carcinoma (BCC) or squamous cell carcinoma (SCC) of the skin, carcinoma in situ of the uterine cervix, adequately resected differentiated thyroid cancer, intraepithelial carcinoma of the neck or breast, or prostate cancer that can be monitored for progress status without any treatment)

9. Hypersensitivity to the investigational products

10. Peripheral neuropathy ≥ Grade 2

11. Pregnant or nursing women

12. Human immunodeficiency virus (HIV)-positive

13. Active hepatitis B or hepatitis C infection
